# From Heart Murmur to Bone Involvement: Tricuspid Valve Endocarditis With First-Rib Osteomyelitis in a Patient With Methicillin-Resistant Staphylococcus aureus (MRSA) Bacteremia

**DOI:** 10.7759/cureus.116470

**Published:** 2026-09-18

**Authors:** Tanner Besse, Rodolfo Loureiro, Nicholena Richardson, Shayne Gue

**Affiliations:** 1 Emergency Medicine, St. Joseph’s Hospital, BayCare Health System, Tampa, USA

**Keywords:** addiction and infectious disease intersection, emergency medicine, infective endocarditis, intravenous drug user, osteomyelitis

## Abstract

Tricuspid valve infective endocarditis (TVIE) is most commonly associated with intravenous drug use and *Staphylococcus aureus* bacteremia. While right-sided endocarditis may present with septic pulmonary emboli, hematogenous spread to the bone is a rare but serious complication. Among osseous sites, first-rib osteomyelitis is particularly uncommon and may be underrecognized in patients presenting with localized chest pain or swelling.

We present the case of a 37-year-old woman with intravenous substance use who presented to the emergency department with left-sided upper chest pain, swelling, and erythema for two weeks. The patient was tachycardic on arrival. Laboratory evaluation revealed anemia, eosinophilia, and elevated erythrocyte sedimentation rate (ESR) and C-reactive protein (CRP) levels. Computed tomography (CT) of the chest revealed findings consistent with first-rib osteomyelitis with phlegmon formation. The patient received initial antibiotic therapy in the emergency department, followed by admission for intravenous (IV) antibiotic administration and Infectious Disease consultation. This case highlights the importance of prompt recognition of osteomyelitis involving uncommon osseous structures to prevent further systemic complications and facilitate timely antimicrobial and surgical management.

## Introduction

Right-sided native valve infective endocarditis (RSIE) involves either the tricuspid valve (TVIE) or the pulmonic valve (PVIE). Isolated RSIE accounts for up to 10% of cases of infective endocarditis and is mainly encountered in populations with intravenous drug use [1]. The most common pathogen is *Staphylococcus aureus*. RSIE usually presents with persistent fever and respiratory symptoms, while signs of systemic embolization, as seen in left-sided infective endocarditis, are notably absent [1]. The respiratory symptoms are typically secondary to septic pulmonary emboli, which occur in approximately 53% of patients with TVIE and include cough, pleuritic chest pain, hemoptysis, and dyspnea [2].

Osteomyelitis is an infection of the bone, with an incidence of 1 in 675 hospital admissions in the United States each year, or approximately 50,000 cases annually [3]. Bone infection may result from bacteremic seeding from a distant source of infection, contiguous spread from surrounding tissues or joints, or direct inoculation following trauma or surgery [4]. In adults, hematogenous osteomyelitis most commonly affects the vertebrae, whereas in children, the metaphysis of the long bones is most commonly affected [5]. Osteomyelitis of flat bones is exceedingly rare, and osteomyelitis of the ribs accounts for less than 1% of all cases [6]. The diagnosis of osteomyelitis requires a thorough history and physical examination, with a high degree of clinical suspicion being particularly important. Patient factors that should increase suspicion include a history of intravenous drug use, nonhealing wounds, orthopedic hardware or implants, and documented recent bacteremia. These features, combined with the classic signs and symptoms of osteomyelitis, should prompt an astute emergency medicine physician to consider the diagnosis. Osteomyelitis usually presents with a gradual onset of dull pain at the involved site, with local findings such as tenderness, warmth, erythema, and swelling often present. Notably, systemic symptoms such as fever and chills may be absent in isolated cases of osteomyelitis. These systemic symptoms are more common in the presence of concurrent bacteremia or soft-tissue infection.

This case highlights the importance of maintaining a high level of clinical suspicion for osteomyelitis when patients with typical risk factors present with atypical osseous involvement.

## Case presentation

Patient information

A 37-year-old woman with a past medical history of polysubstance use, active hepatitis C infection, methicillin-resistant *Staphylococcus aureus* (MRSA) bacteremia, TVIE (status post AngioVac extraction in February 2025), and septic pulmonary emboli presented to the emergency department with a chief complaint of left upper chest pain of two weeks’ duration. She reported that the pain was progressive and accompanied by swelling, erythema, and mild shortness of breath. She denied fever, chills, nausea, vomiting, or other localized pain. The patient reported that she had been discharged one month earlier following a six-day admission for a “mass on (her) heart.” Upon review of her medical records, it was found that she had been diagnosed with MRSA bacteremia with subsequent TVIE and septic pulmonary emboli. She was discharged with a plan for prolonged IV antibiotic therapy at an outpatient infusion center. However, due to transportation difficulties, she was unable to attend her scheduled infusion appointments. Since discharge, the patient had continued to use IV amphetamines and fentanyl. 

Clinical findings

On arrival, the patient’s vital signs were as follows: temperature, 98.4 °F (oral); heart rate, 100 beats/minute (peripheral); blood pressure, 117/53 mmHg; respiratory rate, 18 breaths/minute; oxygen saturation, 100%; and body mass index, 19.8 kg/m².

Physical examination revealed a cachectic, chronically ill-appearing woman in no acute distress. She was tachycardic and had a new systolic murmur, along with a tender, erythematous mass overlying the left upper parasternal region. The patient’s neck and bilateral forearms had hyperpigmented scars along accessible veins.

Given the patient’s history and physical examination findings, the initial differential diagnosis included bacteremia, recurrent TVIE, osteomyelitis of the sternum and/or rib, septic arthritis of the left sternoclavicular joint, traumatic chest wall injury, cellulitis, and abscess.

Diagnostic assessment

Complete blood count was significant for a hemoglobin level of 10.9 g/dL, hematocrit of 32.4%, red cell distribution width of 15.3%, platelet count of 485,000/mm³, eosinophils of 10.7%, absolute eosinophil count of 0.8 × 10⁹/L, and erythrocyte sedimentation rate of 42 mm/h. The complete metabolic panel showed an elevated total protein level of 9.1 g/dL and an elevated globulin level of 5.6 g/dL. The patient also had an elevated C-reactive protein level of 9 mg/dL (Table 1).

**Table 1:** Initial laboratory findings

Investigation	Value	Reference range
Hemoglobin	10.9 g/dL	12.0-16.0 g/dL
Hematocrit	32.40%	37.0%-47.0%
Red cell distribution width	15.30%	11.5%-14.5%
Platelets	485 thousand/uL	140-450 thousand/uL
Eosinophils	10.70%	0.0%-6.0%
Eosinophils, absolute	0.8 thousand/uL	0.0-0.4 thousand/uL
Erythrocyte sedimentation rate	42 mm/hr	0-20 mm/hr
Protein, total	9.1 g/dL	6.4-8.2 g/dL
Globulin	5.6 g/dL	2.4-4.2 g/dL
C-reactive protein	9.00 mg/dL	0.00-0.50 mg/dL
High-sensitivity troponin	<3 ng/L	≤14 ng/L
BN peptide	<10.0 pg/mL	0.0-100.0 pg/mL

Given the patient’s chief complaint of chest pain, an electrocardiogram was performed, which demonstrated normal sinus rhythm at a rate of 92 beats/minute, with no ST-segment changes and normal intervals. Plain chest radiographs were also obtained to assess the osseous structures of the thorax. The radiographs demonstrated findings suspicious for trace pleural fluid, a 3.6-cm mass-like opacity in the left upper lobe, and a normal cardiac silhouette without pneumothorax (Figure 1). Given the clinical suspicion for osteomyelitis, computed tomography (CT) of the chest was subsequently performed (Figure 2 and Figure 3). CT demonstrated a heterogeneous mass surrounding the abnormal anterior left first rib, which was concerning for osteomyelitis with surrounding phlegmon and worsening osseous destruction of the anterior left first rib. There was no evidence of a drainable abscess or involvement of the left sternoclavicular joint. Additionally, sequelae of the patient’s prior septic pulmonary emboli were noted on both chest radiography and CT.

**Figure 1:**
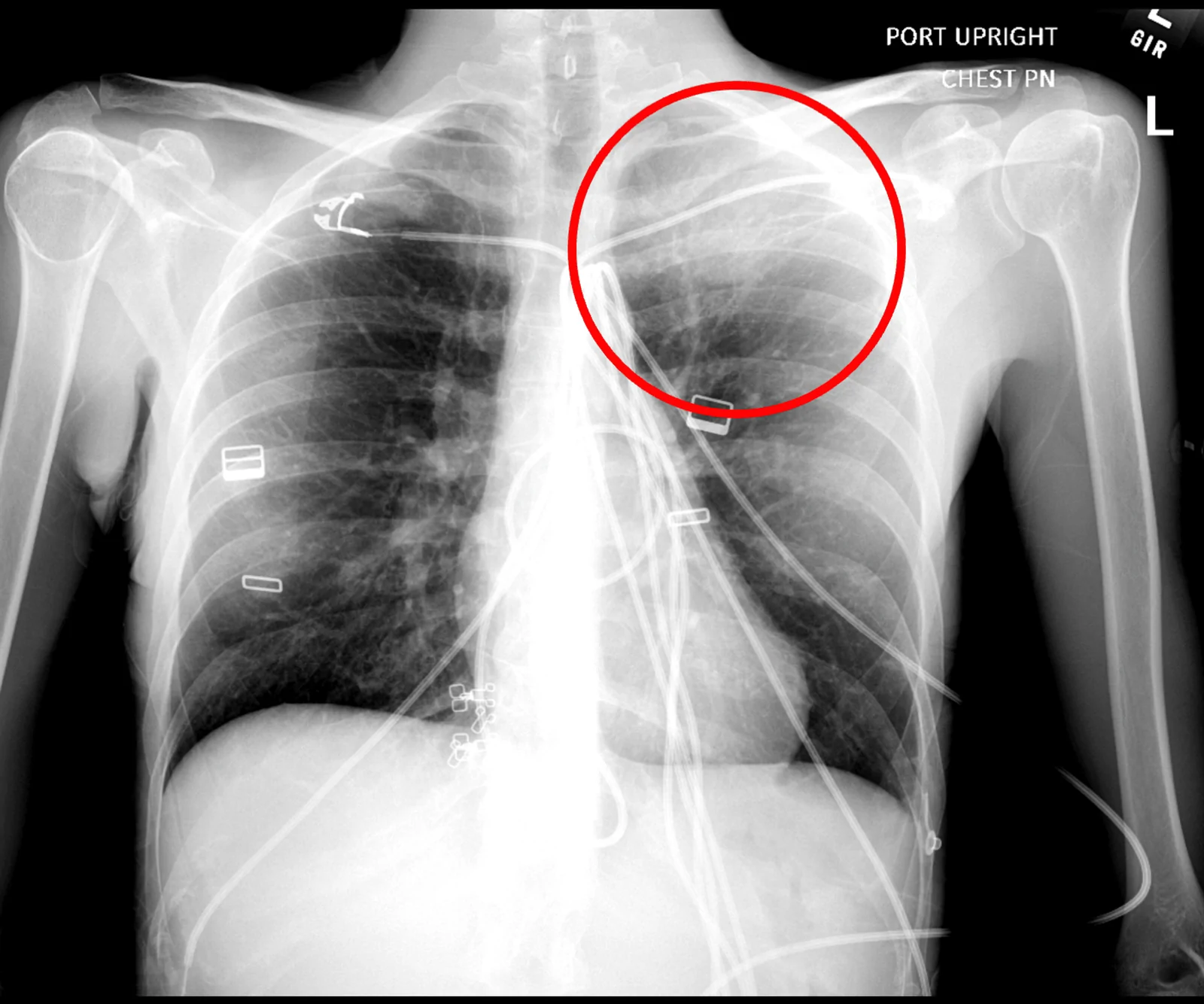
Upright chest radiography demonstrating a 3.6-cm mass-like opacity in the left upper lung lobe (red circle) with a normal cardiac silhouette and no pneumothorax

**Figure 2:**
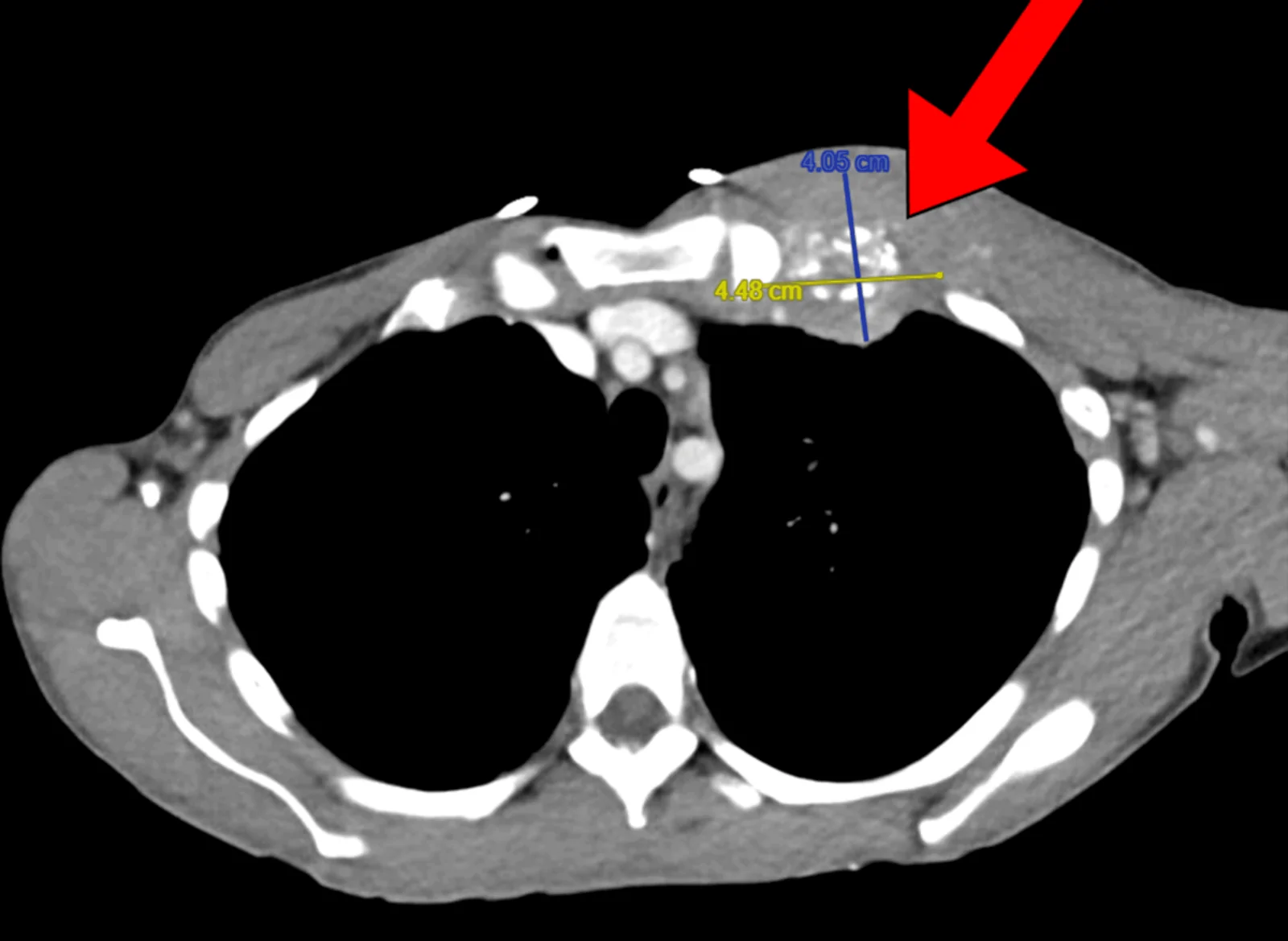
Axial CT of the chest demonstrating lytic destruction of the first rib (red arrow)

**Figure 3:**
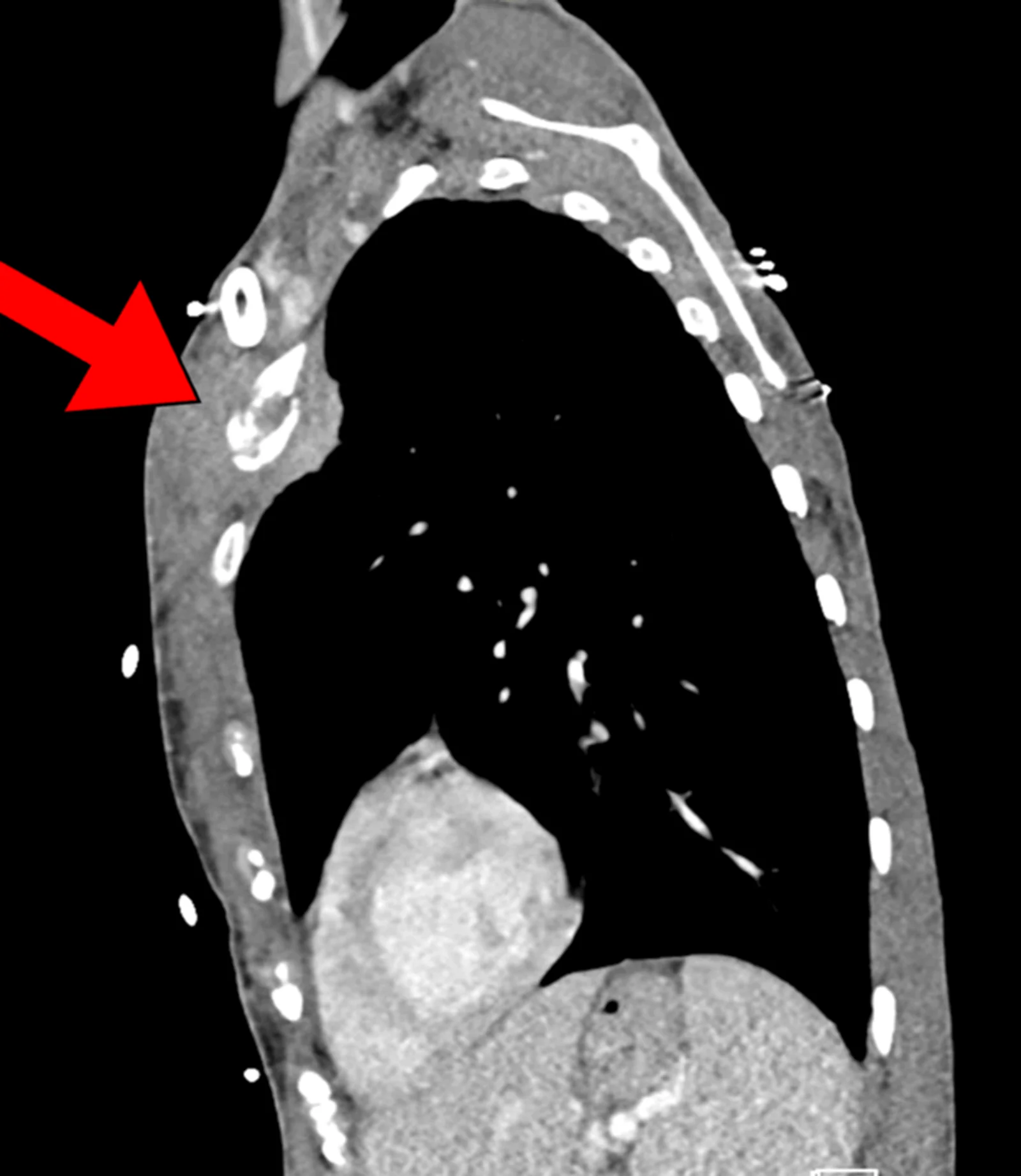
Sagittal CT of the chest demonstrating lytic destruction of the first rib (red arrow)

Follow-up and outcomes

Based on the patient’s history, physical examination findings, laboratory results, and diagnostic imaging, she was preliminarily diagnosed with presumed left first-rib osteomyelitis in the setting of recent MRSA bacteremia and TVIE. Blood cultures were obtained in the emergency department, and the patient received a 1,250-mg dose of IV vancomycin. She was admitted to the medical service for inpatient IV antibiotic therapy. On admission, daptomycin (350 mg/50 mL) was scheduled at a dose of 267.6 mg every 24 hours.

The patient remained hospitalized for four days, during which no adverse events occurred. The blood cultures obtained in the emergency department showed no growth after five days. During her inpatient stay, she was evaluated by the Infectious Disease service, which recommended discharge on oral doxycycline 100 mg twice daily for 4 weeks. She was also instructed to follow up with her infectious disease provider and was provided with resources to establish care with a primary care provider at a local clinic. Since this encounter, the patient has not returned to our emergency department.

## Discussion

IV drug use is associated with numerous complications, one of the most serious being bacteremia, which can lead to a range of distant infections. The most common pathogen, MRSA, can spread through the venous circulation to the heart, creating a nidus of infection, most commonly involving the tricuspid valve [1]. The diagnosis of infective endocarditis is based on the Duke criteria, originally described in 1994 [7]. Overall mortality in native-valve infective endocarditis is approximately 6%, and this rate, particularly among people who inject drugs (PWID), has been associated with vegetation size [8].

Once TVIE is established, septic pulmonary emboli can develop, resulting in pulmonary symptoms. In many cases, the infection remains localized to the pulmonary circulation, with patients experiencing dyspnea, pleuritic chest pain, cough, and hemoptysis [2].

In this case, systemic hematogenous spread occurred, a complication more commonly associated with left-sided than right-sided infective endocarditis. One potential site of systemic dissemination is the bone, leading to osteomyelitis. The most common sites of osteomyelitis are the vertebrae in adults and the metaphyses of long bones in children [5]. The significance of this case lies in the involvement of the ribs, an uncommon site of osteomyelitis [6]. When the rib is involved, the most common presenting finding is localized chest wall swelling; therefore, the diagnosis can mimic soft-tissue infection, abscess, or even malignancy [9].

The diagnosis of osteomyelitis is supported by laboratory findings of elevated inflammatory markers, including CRP and ESR. Radiographic imaging plays a crucial role in the evaluation of patients with suspected osteomyelitis. The most valuable modalities include plain radiography, MRI, and technetium-99 bone scans [5]. Additionally, CT is useful for assessing the extent of bony and soft-tissue involvement and can guide percutaneous needle aspiration or biopsy [10]. Bone biopsy, whether open or percutaneous, is critical for confirming the histopathological diagnosis of osteomyelitis, identifying the causative pathogen, and obtaining antibiotic susceptibility data to guide targeted therapy. Given our patient’s recent admission for MRSA bacteremia and TVIE, additional confirmatory testing was deferred per the inpatient team’s recommendation.

The treatment of osteomyelitis hinges on identification of the causative pathogen and susceptibility testing, followed by prompt administration of appropriate antibiotics. Surgery also plays an important role in management. The early combination of prolonged antibiotic therapy and surgical intervention is associated with favorable outcomes in acute osteomyelitis; however, recurrence is not uncommon [11]. Antibiotic selection should be guided by susceptibility testing. When MRSA is suspected, empiric treatment consists of IV vancomycin; alternatively, linezolid may be used, including via the oral route because of its high oral bioavailability and approximately 1:1 oral-to-IV dose equivalence. The duration of antibiotic therapy is generally six weeks for bacterial osteomyelitis [10]. Given our patient’s recent unsuccessful course of outpatient IV antibiotic therapy related to social determinants of health, oral antibiotic therapy was selected. Surgical consultation is an important component of osteomyelitis management and is generally recommended in cases of persistent or recurrent bloodstream infection or worsening pain despite appropriate medical therapy [10]. This recommendation is particularly strengthened by the presence of abscess formation or evidence of necrosis. Osteomyelitis remains a serious clinical condition, with reported in-hospital mortality rates ranging from 1.7% to more than 27% in cases complicated by septicemia [12].

## Conclusions

First-rib osteomyelitis in the setting of MRSA bacteremia and TVIE is a rare but severe manifestation of hematogenous seeding in high-risk patients. Emergency clinicians play a critical role in recognizing atypical presentations of endocarditis-related complications and initiating prompt antimicrobial therapy. This case highlights the importance of thorough re-evaluation following incomplete outpatient treatment and underscores the impact of social barriers on infection recurrence. Prompt diagnosis and appropriate management can improve patient outcomes and prevent complications. This case also emphasizes the importance of maintaining a broad differential diagnosis when evaluating painful chest wall masses and using the patient’s clinical history to recognize otherwise unusual diagnoses. These principles can help prevent complications of osteomyelitis, including permanent osseous damage, further hematogenous or contiguous spread, worsening sepsis, and death.
